# Continuous Intra-Arterial Blood Pressure Monitoring Improves the Efficiency of Percutaneous Balloon Compression of the Trigeminal Ganglion for Trigeminal Neuralgia

**DOI:** 10.1155/2022/7567630

**Published:** 2022-10-03

**Authors:** Yuchao Zuo, Dengpan Song, Yan Hu, Shengqi Zhao, Mingchu Zhang, Meng Wang, Fuyou Guo

**Affiliations:** ^1^Department of Neurosurgery, First Affiliated Hospital of Zhengzhou University, No. 1 Jianshe East Road, Zhengzhou, Henan Province, China; ^2^International Joint Laboratory of Nervous System Malformations, First Affiliated Hospital of Zhengzhou University, No. 1 Jianshe East Road, Zhengzhou, Henan Province, China

## Abstract

**Objective:**

The aim of the study is to explore the characteristics of systolic blood pressure (SBP) and heart rate (HR) changes in patients undergoing percutaneous balloon compression (PBC) for trigeminal neuralgia and analyze the factors that influence the formation of the symbolic pear shape of the balloon, which signifies successful compression.

**Methods:**

We retrospectively analyzed the changes in systolic blood pressure (SBP) and heart rate (HR) in 103 consecutive patients with trigeminal neuralgia (TN). Fifty-five patients who underwent operations with intermittent inflating cuff blood pressure (IICBP) monitoring were classified into the IICBP group. The other 48 patients who underwent continuous intra-arterial blood pressure (CIABP) monitoring were classified into the CIABP group.

**Results:**

Among all the patients, there were more women than men and the patients in both the groups more commonly had TN on the right side and involving branch III. First, the balloon appeared as “pear-shaped” when the compression was effective, and the SBP increased an average of 59.04% in the CIABP group. CIABP provided us the precise range of the increase. Older patients had higher SBPs, especially patients with hypertension. Second, SBP was more sensitive and lasted much longer than HR in the process of puncturing the foramen ovale and compressing the ganglion. SBP fluctuated much more in the CIABP group than in the IICBP group. Third, the median time taken in the CIABP group was less than the IICBP group, and the prognosis of the CIABP group was better than the IICBP group.

**Conclusion:**

Effective ganglion compression significantly increased SBP. CIABP can monitor SBP in real time and can also safeguard the compression process. CIABP is a safe and effective method in the PBC process that is worthy of promotion and application.

## 1. Introduction

Trigeminal neuralgia (TN) is one of the most severe facial pain syndromes [1–4]. Pharmacological interventions cannot provide long-term pain relief, and microvascular decompression, percutaneous balloon compression (PBC), gamma knife radiotherapy, radiofrequency thermocoagulation, glycerol rhizotomy, and other surgeries have been useful for pain control in primary TN [5–8].

Among these surgical options, PBC is a commonly used microinvasive technique and shows potential in the control of TN [9, 10]. At present, the pear shape of the balloon is the gold-standard indicator of effective compression and the final successful indicator [8, 11–14]. The process of forming the “pear” is the most critical and central step. We cannot see whether the balloon catheter has punctured into Meckel's cave intraoperatively. Clinicians have long tried to find indicators of correct puncturing of Meckel's cave and to find new techniques that can improve the operative efficiency [6, 9, 10, 13–16].

Repeating an incorrect cannulation may hinder success and even cause severe complications [11, 12]. Moreover, it is difficult to obtain a pear shape in patients with damaged Meckel's caves or pseudopuncture channels. This study retrospectively analyzed the characteristics of systolic blood pressure (SBP) and cardiac rhythm in 114 consecutive patients with TN in our institution.

## 2. Methods

### 2.1. Patient Population

**Figure 1:**
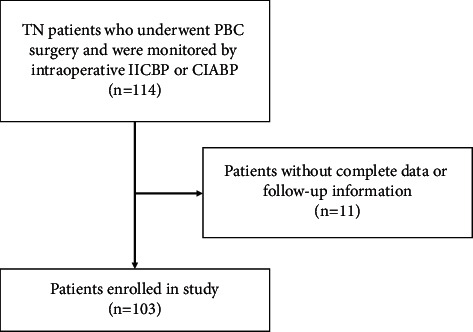
Patient selection process in this study.

After obtaining the approval of the Ethics Review Committee of the First Affiliated Hospital of Zhengzhou University, the medical records and follow-up data were retrospectively collected and analyzed for all patients who were diagnosed with TN and had surgery at the hospital from October 2018 to December 2021. The following inclusion criteria were used: (1) patients older than 18 years old; (2) patients with craniofacial pain with TN characteristics who received a clinical diagnosis of TN before surgery; (3) patients who underwent PBC of the trigeminal ganglion for the treatment of TN with intraoperative intermittent inflating cuff blood pressure (IICBP) or continuous intra-arterial blood pressure (CIABP) monitoring; and (4) patients all underwent effective ganglion compression. The balloon appeared as a “pear” in 12 patients with cardiopathic disease and 35 patients with hypertension. Patients whose detailed perioperative clinical data or follow-up data were incomplete were excluded. The specific screening process is shown in Figure 1. Ultimately, data from 103 patients were included. Informed consent was obtained from all the patients.

### 2.2. PBC Procedures

#### 2.2.1. IICBP Monitoring Procedure

**Figure 2:**
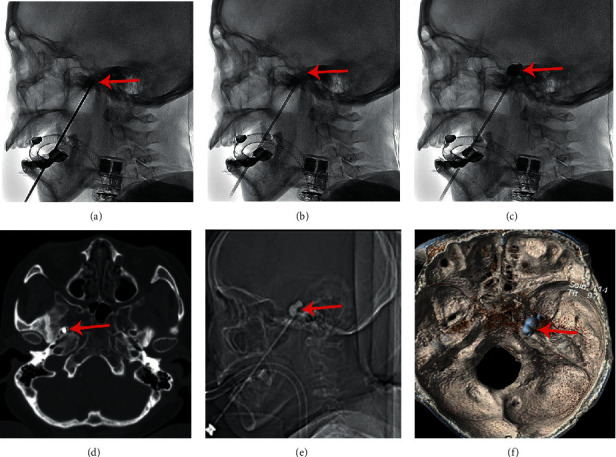
Imaging of the surgical process. (a–c) By the C-arm in the IICBP group and (d–f) in the CIABP group by CT. (a), (d) Puncturing into the FO (red arrow). (b) Introducing the catheter (red arrow) into Meckel's cave. (c), (e) Inflating the balloon as a pear shape (red arrow) imaging laterally. (f) 3D-construction of the pear-shaped balloon on the CT scan (the red arrow pointing to the neck of the balloon).

Sixty-four patients with an IICBP monitoring procedure underwent surgeries under general anesthesia via a tracheal intubation or laryngeal mask. SBP was routinely measured by an intermittent inflating cuff at 3 min intervals. The skin was punctured 2.5 cm lateral and 0.5 cm superior to the commissure of the lips, and the other two reference points were 3 cm anterior to the external auditory meatus along the zygomatic arch and 1 cm inferior to the pupil [6]. Usually, there were three major stages. The first stage was puncturing into the foramen ovale (FO). Guided by the C-arm/CT X-ray, the needle was placed into the FO using the classical Hakanson's technique [17]. When this placement was confirmed by imaging (Figure 2(a)), the precise measurements of SBP and HR in the basal condition (before puncture) and after puncturing into FO were recorded by the anesthesiologist independently. Second, the catheter was introduced into Meckel's cave (Figure 2(b)). Sometimes, puncturing the FO does not mean that the needle is correctly placed in Meckel's cave. Guided by the C-arm/CT X-ray, we repeatedly adjusted the direction and depth of the needle until the position of the catheter's tip was identified by using a C-arm image intensifier in the lateral views; the position of the catheter tip was checked with respect to neighboring bone landmarks [6, 18, 19]. The core of the needle was withdrawn, and a Fogarty balloon catheter (Shenzhen qingyuan) was introduced through the needle and pushed 1.2 to 1.4 cm beyond the needle [17]. Third was inflating the balloon to compress the ganglion. The core of the catheter was pulled out and 0.4 to 1.0 ml of Omnipaque, a contrast agent, was slowly injected into the catheter to inflate the balloon until the fluoroscopic pear shape, which represents effective compression, appeared (Figure 2(c)). The neck of the balloon was right in the trigeminal notch, and the precise measurements of SBP and HR during this period were monitored. After 3 to 8 min of ganglion compression, the balloon was deflated and withdrawn.

#### 2.2.2. CIABP Monitoring Procedure

The other 50 patients in the CIABP group underwent surgery under general anesthesia with CIABP monitoring. Prior to the induction of general anesthesia, the intra-arterial catheter was inserted using ultrasound into the radial artery and was connected to a disposable pressure transducer with standard low-compliance tubing, and the arterial pressure was continuously recorded using an electrocardiogram monitor. CIABP monitoring was performed.

Like IICBP, CIABP had three major procedures. First, the needle was initially fluoroscopically directed to the FO (Figure 2(d)), and the SBP and HR at this period were recorded by the anesthesiologist independently. Then, the core of the needle was withdrawn, introducing the catheter into Meckel's cave. Finally, we inflated the balloon/compressed the ganglion, and the ideal pear shape was achieved (Figures 2(e)–2(f)), which meant that Meckel's cave was fully covered. The anesthesiologist recorded the SBP and HR at the stage of effective ganglion compression (pear shape of the balloon). The balloon was deflated after compression.

### 2.3. Follow-Up and Statistical Analysis

A total of 103 patients were followed up for a mean of 18.7 months (range of 2 months to 30 months). There were no deaths and no patients had severe complications (hemiplegia, hemorrhage, loss of sight, diplopia, or facial paralysis). Ten patients suffered from herpes. Analyses were performed with the IBM SPSS 26 Statistics. For measurement data, if the variables followed a normal distribution, the means and standard deviations were calculated, and a *t* test or analysis of variance was used for the intergroup comparisons. For nonnormal distributions, the variables are expressed as medians and interquartile ranges (IQRs), quartiles were calculated, and the Mann–Whitney *U* test or Kruskal–Wallis H test was used for the intergroup comparisons. For categorical data, frequencies and percentages were calculated, and the chi-squared test was used for the intergroup comparisons. The recurrence-free survival of both groups was estimated and compared using the Kaplan–Meier method. Values of *p* < 0.05 were considered significantly.

## 3. Results

### 3.1. Demographic Data

A total of 55 patients received IICBP monitoring and 48 patients received CIABP monitoring. The mean ages of the two groups were 58.8 years (IICBP group) and 61.1 years (CIABP group) (*p* = 0.354), respectively. There was no significant difference in the sex distribution between the two groups (*p* = 0.772), and there were more women than men. Similarly, there was no significant difference between the two groups in the location of TN (*p* = 0.767) or the specific nerve involved (*p* = 0.315), and the patients in both the groups more commonly had TN on the right side and involving branch III **(**Table 1**)**. Before referral to our hospital, 7 patients received microvascular decompression (MVD) (3 in the IICBP group and 4 in the CIABP group), and 4 underwent percutaneous radiofrequency thermocoagulation or glycerol rhizotomy (1 in the IICBP group and 3 in the CIABP group).

### 3.2. Changes in HR in the PBC Procedure

**Figure 3:**
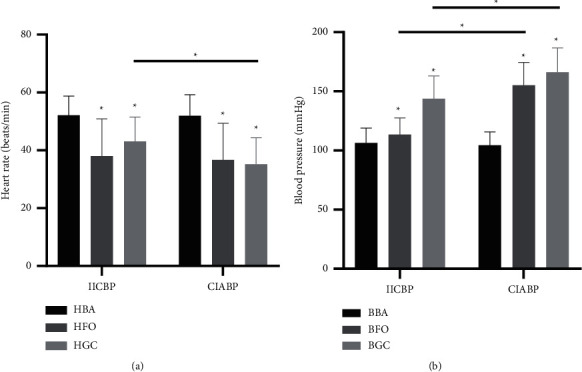
HR and SBP in patients undergoing operations for TN by means of PBC under general anesthesia with intubation. Values are given for the basal condition (BA), immediately after FO puncture (FO), during effective ganglion compression (GC). (a) HR change. (b) SBP change. HBA : HR of basal conditions before puncture; HFO: the lowest HR after FO puncture; HGC: the lowest HR during effective ganglion compression; BBA : SBP under basal conditions before puncture; BFO: the highest SBP after FO puncture; BGC: the highest SBP during effective ganglion compression. (^*∗*^*p* < 0.05).

The HR changes during PBC treatment were monitored and statistically analyzed in both the groups. There was no difference in the baseline HR between the two groups before the operation after anesthesia (*p*=0.925); however, the HR of the patients immediately decreased after the needle entered the FO (HFO) in both the groups (*p* < 0.001). When effective ganglion compression was performed, the HR of the ganglion compression period (HGC) remained lower than the baseline HR in both the groups (*p* < 0.001), but HGC decreased by an average of 32.38% in the CIABP group, which was significantly more than in the IICBP group (*p* < 0.001) **(**Figure 3(a)).

### 3.3. Changes in SBP in the PBC Procedure

Similar to the HR changes, the SBP changes during PBC treatment were monitored and statistically analyzed in both the groups. There was no difference in the BBA between the two groups before the operation after anesthesia (*p*=0.384). The BFO of both the groups immediately increased (*p* < 0.001), but the BFO increased by an average of 48.59% in the CIABP group, which was significantly greater than in the IICBP group (*p* < 0.001). The BGC remained higher than the BBA in both the groups (*p* < 0.001), but the BGC also increased by an average of 59.04% in the CIABP group, which was significantly more than in the IICBP group (*p* < 0.001) **(**Figure 3(b)**)**.

### 3.4. Comparison between HR and SBP

**Figure 4:**
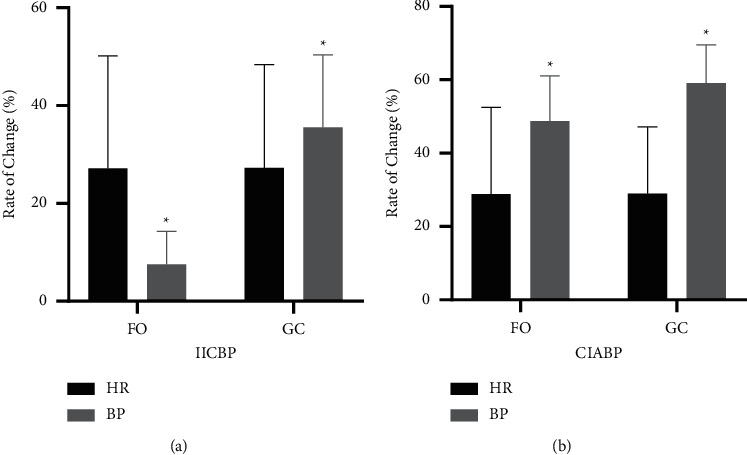
The rate change of the heart rate (HR) and systolic blood pressure (BP) in the stage of puncturing the foramen ovale (FO) and effective ganglion compression (GC). (a) IICBP group. (b) CIABP group. (^*∗*^*p* < 0.05.)

To explore the sensitivity of HR and SBP, we further analyzed the magnitude of all changes in HR and SBP. All values were compared with the baseline to obtain the rates of change. The results showed that in the IICBP group, the change rate of the SBP at the FO puncture stage was lower than that of the HR (*p* < 0.001), while the change rate of the SBP at the ganglion compression stage was significantly higher than that of the HR (*p* < 0.001). In the CIABP group, the SBP change rate at the FO puncture stage and effective ganglion compression stage was significantly higher than the HR change rate (*p* < 0.001) **(**Figure 4**)**.

### 3.5. Operation Time of PBC

**Figure 5:**
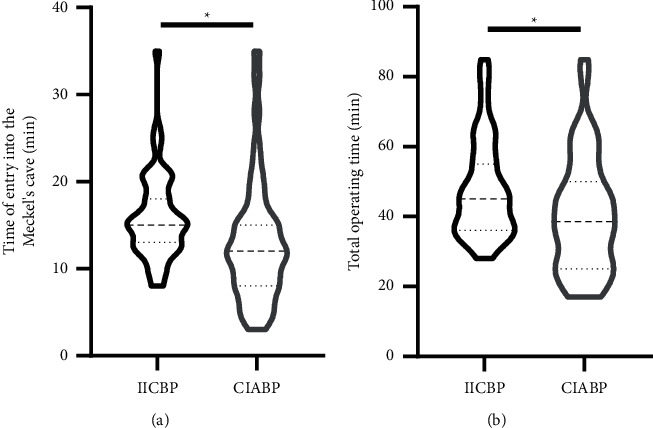
Comparison of the time of entry into Meckel's cave and the total operating time between the two groups. (a) The time of entry into Meckel's cave. (b) The total operating time. (^*∗*^*p* < 0.05).

To study the difference in the operation time between the two monitoring methods, the time of entry into Meckel's cave and the total operating time were statistically analyzed. The results showed that the median time to enter Meckel's cave in the CIABP group was 12 min (IQR: 8 min–15 min), while that in the IICBP group was 15 min (IQR: 13 min–18 min). Similarly, the median time of the whole operation was 38.5 min (IQR: 25 min–50 min) in the CIABP group and 45 min (IQR: 36 min–55 min) in the IICBP group. Therefore, the time of entry into Meckel's cave (*p*=0.011) and the total operating time (*p*=0.0134) in the CIABP group were significantly less than those in the IICBPP group **(**Figure 5).

### 3.6. Postoperative Effects

**Figure 6:**
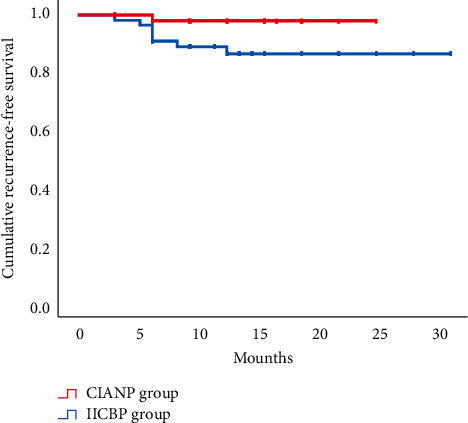
Kaplan–Meier curves of the recurrence-free survival of both the groups.

All patients underwent the CT scans before the operation, and they benefited from the careful assessment because, overall, the FO was successfully punctured. Postoperatively, initial pain relief was achieved in 49 (89.09%) patients in the IICBP group and 47 (97.92%) in the CIABP group (*p*=0.167). We performed follow-up statistics on the postoperative complications. The median follow-up time was 12 months in both the groups (*p*=0.223). The most common postoperative short-term complication was facial numbness, with an incidence of 50.90% in the IICBP group and 47.92% in the CIABP group (*p*=0.762). Other complications, such as hypoesthesia (*p*=0.970), masseter weakness (*p*=1.000), and herpes (*p*=0.852), did not differ in incidence between the two groups. Similarly, there was no significant difference in the BNI (Barrow Neurological Institute) facial numbness score between the two groups (*p*=0.584), though the postoperative BNI pain score in the CIABP group was significantly lower than that in the IICBP group (*p*=0.035) **(**Table 2**)**. This finding reflects that the postoperative effect of the CIABP group was better than that of the IICBP group. Among the patients with successful attempts, there were no significant differences in the incidence of common compression-related complications between the groups. Kaplan–Meier curves of the recurrence-free survival of both the groups showed that the prognosis of the CIABP group tended to be better than that of the IICBP group (*p*=0.052**(**Figure 6**)**.

## 4. Discussion

Because of its minimal invasiveness, quick recovery after surgery, and immediate pain relief, PBC is gradually becoming an attractive choice for the treatment of TN, especially in those patients who refuse to undergo open surgery [20, 21]. PBC is an effective and safe option for the elderly population and patients with recurrence after MVD or other unsuccessful treatment [22].

Valid compression of the ganglion is the key factor in success, which can be judged by the shape of balloon [23]. The pear-shaped balloon, which indicates the correct compression of the ganglion, is an important predictor of a successful operation [11]. However, the procedure is performed under general anesthesia, and a variety of factors may influence the compression. We cannot be sure whether the balloon catheter has punctured into Meckel's cave intraoperatively, and it is difficult to evaluate the process [8, 11, 15, 18], especially for new surgeons and puncturing into the wrong location multiple times will ultimately result in failure. Thus, it is critical to find another reliable and useful indicator to minimize surgical complications.

Although guided by the C-arm or CT scan, the puncture is made blindly and has potential risks; the cannulation of the FO and the diversity of the balloon shape all contribute to operative difficulties [24]. Cannulation of the FO is the first step and an important one. Anatomical studies have revealed an association between the size of the FO and TN. The FO puncture is sometimes difficult, especially when the FO is narrow or calcified, which causes difficulty, and the sense of passing through or piercing is deficient. That is, we cannot be sure that the needle has punctured the FO. Occasionally, the foramen lacerum or supraorbital fissure may be mistaken as the FO, and the internal carotid artery or the optic nerves may be injured [11, 15, 25]. However, the CT scans may improve the success rate of puncturing the FO. With the help of the CT scan, we may be sure to enter the FO and may be able to determine when the foramen lacerum or supraorbital fissure has been punctured.

In recent years, different authors performing PBC have reported the occurrence of significant autonomic changes coinciding with the FO puncture and ganglion compression [6, 15, 26]. Under general anesthesia with endotracheal intubation, the HR and SBP all decreased compared to the preanesthetic values. Therefore, if the SBP rapidly increases, there might be confusion regarding the correction compression of the trigeminal ganglion or when puncturing trigeminal nerves. This may be in line with the theory that the mechanical force obtained by the balloon is exerted on the C fibers [6]. In our study, when entering the FO or when compressing the ganglion, SBP increased quickly and significantly; effective compression of the ganglion (pear shape of the balloon) significantly increased the SBP (increased by an average of 59.04%), but there were no significant changes in HR in some patients. Elderly patients or patients with hypertension had a more obvious increase in their SBP. These findings may indicate the advantages of CIABP; CIABP monitoring makes it easier to capture the fluctuation in the process; traditionally, IICBP may miss the severe fluctuations of SBP. In contrast, invalid puncture or unsatisfactory compression (the balloon not taking on a pear or dumbbell shape) will result in a slight increase in the SBP (usually within 40%).

To the best of our knowledge, this is the first study to illustrate the importance of SBP in the PBC process. In previous reports, the authors have usually focused on the decrease in the HR. Generally, we know that using PBC for the FO puncture can elicit bradycardia and can increase SBP, and compression of the ganglion may cause marked tachycardia and can further increase SBP [6, 21]. However, the magnitude of these changes is unknown. Before applying CIABP, the IICBP (not real-time monitoring) responds slower than the HR. In our study, the SBP change was more sensitive and lasted much longer than the HR change in the process of puncturing the FO and compressing the ganglion. During the stage of puncturing the FO, the IICBP group had significant changes in HR and moderate changes in SBP. We hypothesize that this is caused by the limitations of the IICBP monitoring method, which cannot detect and update the changes in SBP over time. In the CIABP group, the variation in SBP in the FO stage exceeded the variation in HR. During the GC stage, the IICBP group had significant changes in HR and SBP (the time of balloon compression was generally greater than 3 min). In the CIABP group, HR and SBP were both monitored in real time, and the change rates of SBP at the FO puncture and at GC were significantly higher than the HR change rate (*p* < 0.001). Significantly, we found that sometimes there was no significant change in HR at the FO puncture or GC stage, but there was a sharp increase in SBP. When SBP increases too much (>200 mmHg), the operation is stopped quickly, which allows for a safe surgical procedure.

The median surgical time with the introduction of CIABP was much shorter than that with traditional IICBP. The use of intraoperative CIABP monitoring may shorten the operation time. The surgical time depended mainly upon the puncturing of the FO/Meckel's cave and the effective compression of the ganglion/balloon to form the pear shape. Puncturing skill, assessment of intraoperative imaging, and SBP/HR monitoring are the important factors in the surgical time. The fluctuations of SBP and HR may provide important information in the process of effective compression.

In the short-term follow-up, the postoperative BNI pain score in the CIABP group was significantly lower than that in the IICBP group (*p*=0.035). This finding shows that the postoperative outcome in the CIABP group was better than that in the IICBP group. Kaplan–Meier curves of the recurrence-free survival of both groups showed that the prognosis of the CIABP group tended to be better than that of the IICBP group (*p*=0.052).

In all, there are three striking findings in our study. First, effective or valid compression (pear shape of the balloon) significantly increased SBP and CIABP provided us with the precise range of the increase. The older the patient was, the higher his or her SBP, especially in patients with hypertension. Second, the SBP change was more sensitive and lasted much longer than the HR change in the process of puncturing the FO and compressing the ganglion. Finally, the median time taken in the CIABP group was less than the IICBP group, and the prognosis of the CIABP group tended to be better than the IICBP group.

This research has limitations. The sample of this study was small and it was a retrospective analysis. CIABP was influenced by other factors (anesthetic drugs, age, hypertension, depth of anesthetic, blood pressure conductor, and so on), and some of the factors could not be kept the same between the groups. Therefore, this study needs to be further verified by multicenter, larger studies, and prospective clinical studies. Whether and to what degree the increase in CIABP is related to the pressure, shape, and location of the balloon all need further investigation.

## 5. Conclusion

Effective ganglion compression significantly increased SBP. Unlike traditional IICBP, CIABP can monitor SBP in real time and can also safeguard the compression process. CIABP, which is more sensitive than HR, is a safe and effective method and is worthy of promotion and application in PBC surgeries.

## Figures and Tables

**Table 1:** Demographic data and conditions of the included patients.

Variables	Total	IICBP	CIABP	*p* Value
N	103	55	48	
Age (years)	59.89 ± 12.33	58.84 ± 12.35	61.1 ± 12.31	0.354
*Sex*				0.772
Female	64	34	31	
Male	39	21	17	
*Sides (n)*				0.767
Left	36	18	19	
Right	65	36	28	
Bilateral	2	1	1	
*Branches affected (n)*				0.315
I	4	2	2	
II	12	9	3	
III	51	22	29	
I + II	7	4	3	
II + III	27	17	10	
I + II + III	2	1	1	

**Table 2:** Summary of the immediate effectiveness and postoperative complications.

	IICBP (*n* = 55)	CIABP (*n* = 48)	*p* value
Follow-up time (months)	12 (9, 15)	12 (9, 15)	0.223
Postoperative immediate pain relief (n)	49	47	0.167
*Postoperative BNI pian score (n)*			0.035
I	41	42	
II	8	5	
III	0	1	
IV	5	0	
V	1	0	
*Complications (n)*			
Facial numbness	28	23	0.762
Hypoesthesia	17	15	0.970
Masseter weakness	4	3	1.000
Herpes	3	4	0.852
BNI facial numbness score (*n*)			0.584
I	27	25	
II	17	18	
III	9	4	
IV	2	1	

## Data Availability

The data used to support the findings of this study are available from the corresponding author upon request via e-mail.
